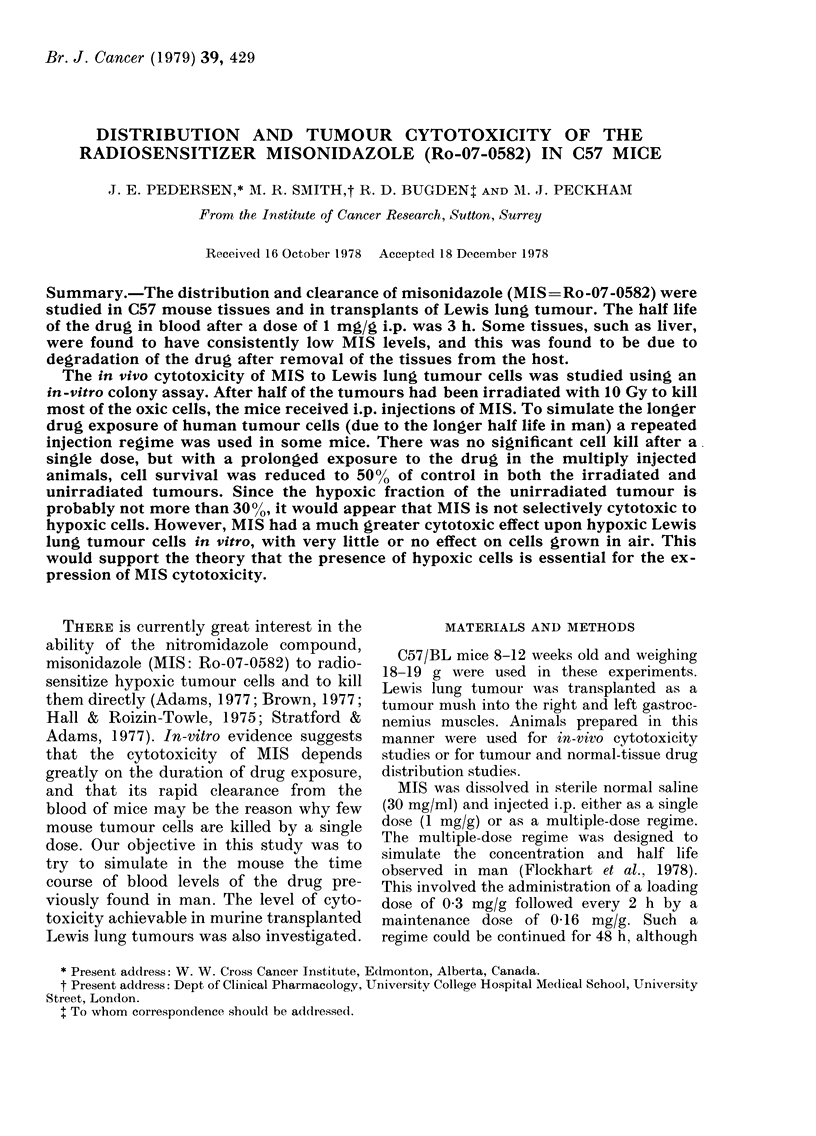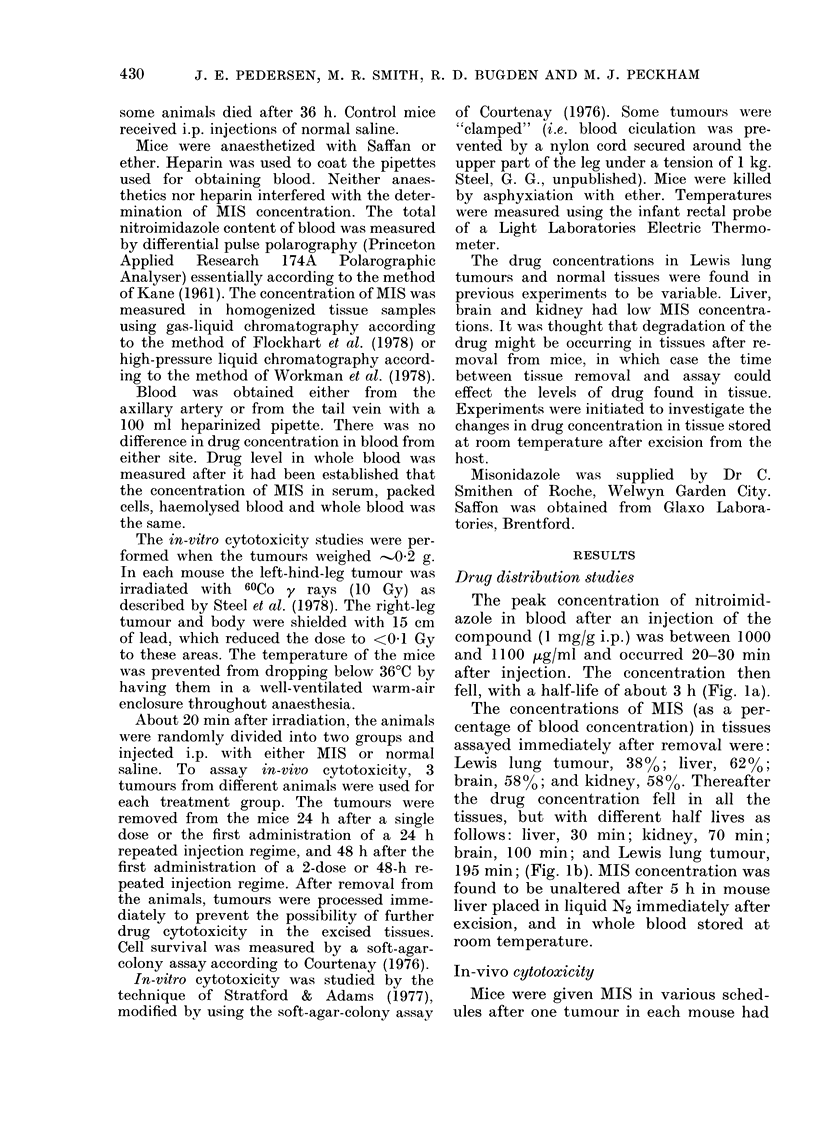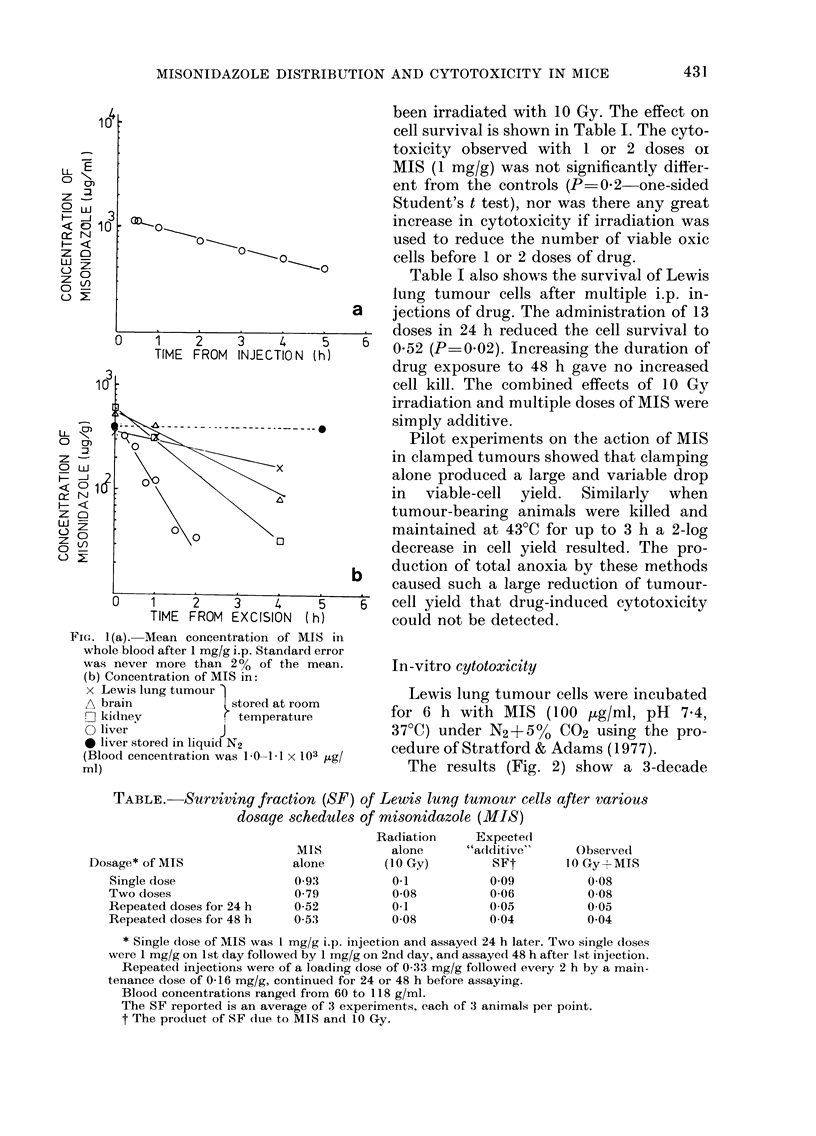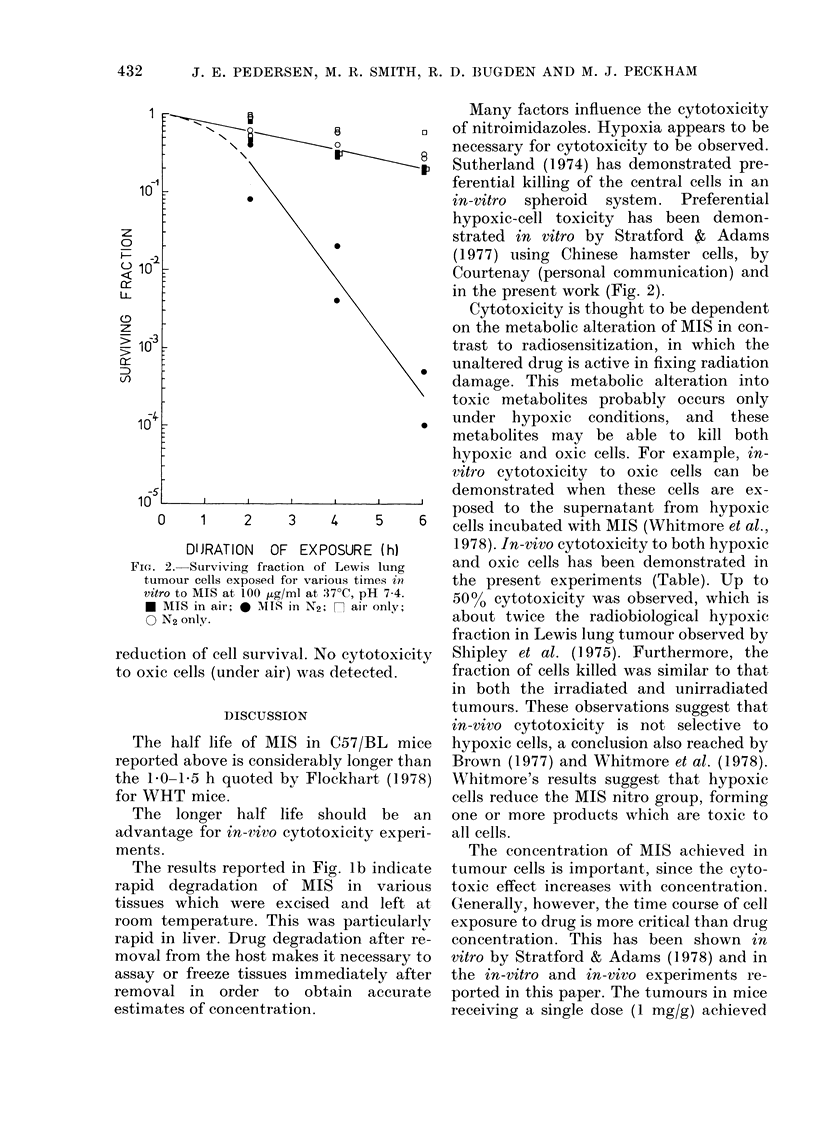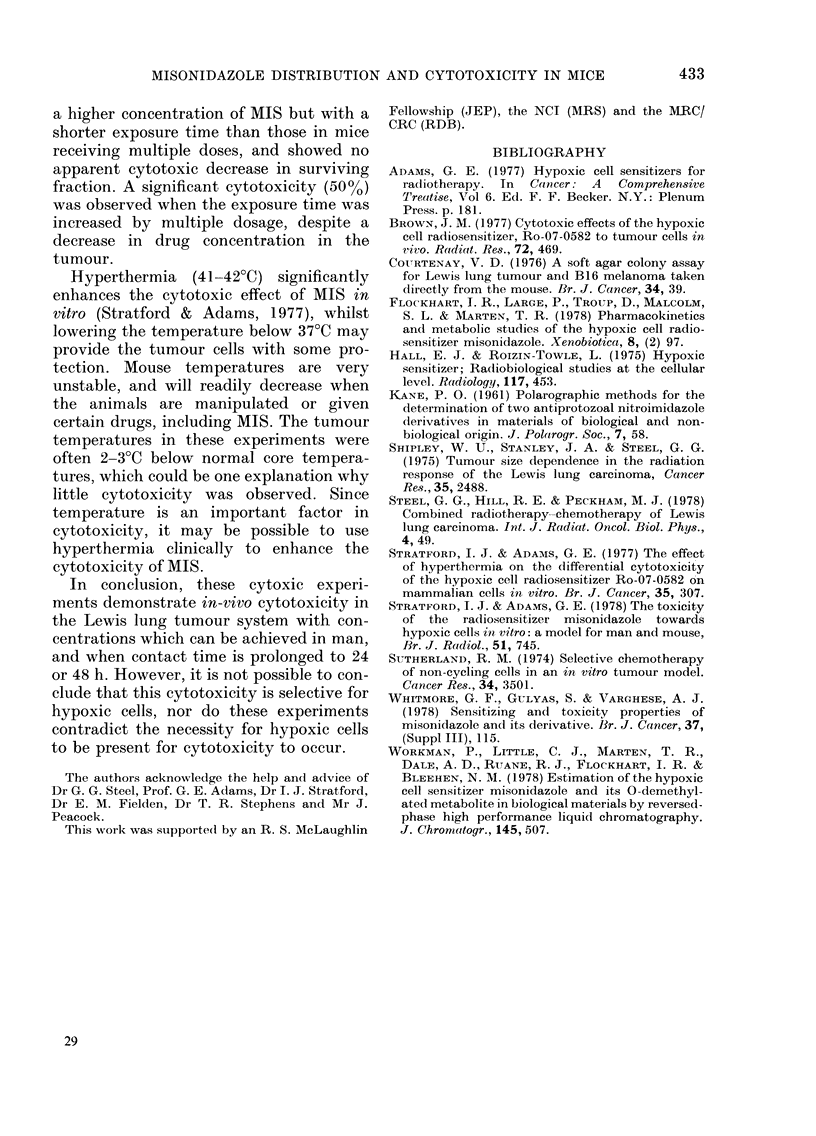# Distribution and tumour cytotoxicity of the radiosensitizer misonidazole (Ro-07-0582) in C57 mice.

**DOI:** 10.1038/bjc.1979.76

**Published:** 1979-04

**Authors:** J. E. Pedersen, M. R. Smith, R. D. Bugden, M. J. Peckham

## Abstract

The distribution and clearance of misonidazole (MIS = Ro-07-0582) were studied in C57 mouse tissues and in transplants of Lewis lung tumour. The half life of the drug in blood after a dose of 1 mg/g i.p. was 3 h. Some tissues, such as liver, were found to have consistently low MIS levels, and this was found to be due to degradation of the drug after removal of the tissues from the host. The in vivo cytotoxicity of MIS to Lewis lung tumour cells was studied using an in-vitro colony assay. After half of the tumours had been irradiated with 10 Gy to kill most of the oxic cells, the mice received i.p. injections of MIS. To simulate the longer drug exposure of human tumour cells (due to the longer half life in man) a repeated injection regime was used in some mice. There was no significant cell kill after a single dose, but with a prolonged exposure to the drug in the multiply injected animals, cell survival was reduced to 50% of control in both the irradiated and unirradiated tumours. Since the hypoxic fraction of the unirradiated tumour is probably not more than 30%, it would appear that MIS is not selectively cytotoxic to hypoxic cells. However, MIS had a much greater cytotoxic effect upon hypoxic Lewis lung tumour cells in vitro, with very little or no effect on cells grown in air. This would support the theory that the presence of hypoxic cells is essential for the expression of MIS cytotoxicity.


*Br. J. Cancer* (1979) **39**, 429

### DISTRIBUTION AND TUMOUR CYTOTOXICITY OF THE RADIOSENSITIZER MISONIDAZOLE (Ro-07-0582) IN C57 MICE

J. E. PEDERSEN,* M. R. SMITH,† R. D. BUGDEN‡ AND M. J. PECKHAM

*From the Institute of Cancer Research, Sutton, Surrey*

Received 16 October 1978 Accepted 18 December 1978

**Summary.—The distribution and clearance of misonidazole (MIS=Ro-07-0582) were studied in C57 mouse tissues and in transplants of Lewis lung tumour. The half life of the drug in blood after a dose of 1 mg/g i.p. was 3 h. Some tissues, such as liver, were found to have consistently low MIS levels, and this was found to be due to degradation of the drug after removal of the tissues from the host.**

**The *in vivo* cytotoxicity of MIS to Lewis lung tumour cells was studied using an *in-vitro* colony assay. After half of the tumours had been irradiated with 10 Gy to kill most of the oxic cells, the mice received i.p. injections of MIS. To simulate the longer drug exposure of human tumour cells (due to the longer half life in man) a repeated injection regime was used in some mice. There was no significant cell kill after a single dose, but with a prolonged exposure to the drug in the multiply injected animals, cell survival was reduced to 50% of control in both the irradiated and unirradiated tumours. Since the hypoxic fraction of the unirradiated tumour is probably not more than 30%, it would appear that MIS is not selectively cytotoxic to hypoxic cells. However, MIS had a much greater cytotoxic effect upon hypoxic Lewis lung tumour cells *in vitro*, with very little or no effect on cells grown in air. This would support the theory that the presence of hypoxic cells is essential for the expression of MIS cytotoxicity.**

THERE is currently great interest in the ability of the nitromidazole compound, misonidazole (MIS: Ro-07-0582) to radiosensitize hypoxic tumour cells and to kill them directly (Adams, 1977; Brown, 1977; Hall & Roizin-Towle, 1975; Stratford & Adams, 1977). *In-vitro* evidence suggests that the cytotoxicity of MIS depends greatly on the duration of drug exposure, and that its rapid clearance from the blood of mice may be the reason why few mouse tumour cells are killed by a single dose. Our objective in this study was to try to simulate in the mouse the time course of blood levels of the drug previously found in man. The level of cytotoxicity achievable in murine transplanted Lewis lung tumours was also investigated.

### MATERIALS AND METHODS

C57/BL mice 8–12 weeks old and weighing 18–19 g were used in these experiments. Lewis lung tumour was transplanted as a tumour mush into the right and left gastrocnemius muscles. Animals prepared in this manner were used for *in-vivo* cytotoxicity studies or for tumour and normal-tissue drug distribution studies.

MIS was dissolved in sterile normal saline (30 mg/ml) and injected i.p. either as a single dose (1 mg/g) or as a multiple-dose regime. The multiple-dose regime was designed to simulate the concentration and half life observed in man (Flockhart *et al.*, 1978). This involved the administration of a loading dose of 0·3 mg/g followed every 2 h by a maintenance dose of 0·16 mg/g. Such a regime could be continued for 48 h, although

* Present address: W. W. Cross Cancer Institute, Edmonton, Alberta, Canada.

† Present address: Dept of Clinical Pharmacology, University College Hospital Medical School, University Street, London.

‡ To whom correspondence should be addressed.

some animals died after 36 h. Control mice received i.p. injections of normal saline.

Mice were anaesthetized with Saffan or ether. Heparin was used to coat the pipettes used for obtaining blood. Neither anaesthetics nor heparin interfered with the determination of MIS concentration. The total nitroimidazole content of blood was measured by differential pulse polarography (Princeton Applied Research 174A Polarographic Analyser) essentially according to the method of Kane (1961). The concentration of MIS was measured in homogenized tissue samples using gas-liquid chromatography according to the method of Flockhart *et al.* (1978) or high-pressure liquid chromatography according to the method of Workman *et al.* (1978).

Blood was obtained either from the axillary artery or from the tail vein with a 100 ml heparinized pipette. There was no difference in drug concentration in blood from either site. Drug level in whole blood was measured after it had been established that the concentration of MIS in serum, packed cells, haemolysed blood and whole blood was the same.

The *in-vitro* cytotoxicity studies were performed when the tumours weighed ~0·2 g. In each mouse the left-hind-leg tumour was irradiated with $^{60}$Co $\gamma$ rays (10 Gy) as described by Steel *et al.* (1978). The right-leg tumour and body were shielded with 15 cm of lead, which reduced the dose to <0·1 Gy to these areas. The temperature of the mice was prevented from dropping below 36°C by having them in a well-ventilated warm-air enclosure throughout anaesthesia.

About 20 min after irradiation, the animals were randomly divided into two groups and injected i.p. with either MIS or normal saline. To assay *in-vivo* cytotoxicity, 3 tumours from different animals were used for each treatment group. The tumours were removed from the mice 24 h after a single dose or the first administration of a 24 h repeated injection regime, and 48 h after the first administration of a 2-dose or 48-h repeated injection regime. After removal from the animals, tumours were processed immediately to prevent the possibility of further drug cytotoxicity in the excised tissues. Cell survival was measured by a soft-agar-colony assay according to Courtenay (1976).

*In-vitro* cytotoxicity was studied by the technique of Stratford & Adams (1977), modified by using the soft-agar-colony assay of Courtenay (1976). Some tumours were "clamped" (*i.e.* blood ciculation was prevented by a nylon cord secured around the upper part of the leg under a tension of 1 kg. Steel, G. G., unpublished). Mice were killed by asphyxiation with ether. Temperatures were measured using the infant rectal probe of a Light Laboratories Electric Thermometer.

The drug concentrations in Lewis lung tumours and normal tissues were found in previous experiments to be variable. Liver, brain and kidney had low MIS concentrations. It was thought that degradation of the drug might be occurring in tissues after removal from mice, in which case the time between tissue removal and assay could effect the levels of drug found in tissue. Experiments were initiated to investigate the changes in drug concentration in tissue stored at room temperature after excision from the host.

Misonidazole was supplied by Dr C. Smithen of Roche, Welwyn Garden City. Saffon was obtained from Glaxo Laboratories, Brentford.

### RESULTS

### *Drug distribution studies*

The peak concentration of nitroimidazole in blood after an injection of the compound (1 mg/g i.p.) was between 1000 and 1100 $\mu$g/ml and occurred 20–30 min after injection. The concentration then fell, with a half-life of about 3 h (Fig. 1a).

The concentrations of MIS (as a percentage of blood concentration) in tissues assayed immediately after removal were: Lewis lung tumour, 38%; liver, 62%; brain, 58%; and kidney, 58%. Thereafter the drug concentration fell in all the tissues, but with different half lives as follows: liver, 30 min; kidney, 70 min; brain, 100 min; and Lewis lung tumour, 195 min; (Fig. 1b). MIS concentration was found to be unaltered after 5 h in mouse liver placed in liquid $N_2$ immediately after excision, and in whole blood stored at room temperature.

### In-vivo *cytotoxicity*

Mice were given MIS in various schedules after one tumour in each mouse had

FIG. 1(a).—Mean concentration of MIS in whole blood after 1 mg/g i.p. Standard error was never more than 2% of the mean. (b) Concentration of MIS in:

× Lewis lung tumour }

△ brain } stored at room

□ kidney } temperature

○ liver }

● liver stored in liquid $N_2$

(Blood concentration was 1·0–1·1 × $10^3$ µg/ml)

been irradiated with 10 Gy. The effect on cell survival is shown in Table I. The cytotoxicity observed with 1 or 2 doses oı MIS (1 mg/g) was not significantly different from the controls ($P=0{\cdot}2$—one-sided Student's $t$ test), nor was there any great increase in cytotoxicity if irradiation was used to reduce the number of viable oxic cells before 1 or 2 doses of drug.

Table I also shows the survival of Lewis lung tumour cells after multiple i.p. injections of drug. The administration of 13 doses in 24 h reduced the cell survival to 0·52 ($P=0{\cdot}02$). Increasing the duration of drug exposure to 48 h gave no increased cell kill. The combined effects of 10 Gy irradiation and multiple doses of MIS were simply additive.

Pilot experiments on the action of MIS in clamped tumours showed that clamping alone produced a large and variable drop in viable-cell yield. Similarly when tumour-bearing animals were killed and maintained at 43°C for up to 3 h a 2-log decrease in cell yield resulted. The production of total anoxia by these methods caused such a large reduction of tumour-cell yield that drug-induced cytotoxicity could not be detected.

### In-vitro *cytotoxicity*

Lewis lung tumour cells were incubated for 6 h with MIS (100 µg/ml, pH 7·4, 37°C) under $N_2$+5% $CO_2$ using the procedure of Stratford & Adams (1977).

The results (Fig. 2) show a 3-decade

TABLE.—*Surviving fraction* (*SF*) *of Lewis lung tumour cells after various dosage schedules of misonidazole* (*MIS*)

| Dosage* of MIS | MIS alone | Radiation alone (10 Gy) | Expected "additive" SF† | Observed 10 Gy+MIS |
|---|---|---|---|---|
| Single dose | 0·93 | 0·1 | 0·09 | 0·08 |
| Two doses | 0·79 | 0·08 | 0·06 | 0·08 |
| Repeated doses for 24 h | 0·52 | 0·1 | 0·05 | 0·05 |
| Repeated doses for 48 h | 0·53 | 0·08 | 0·04 | 0·04 |

* Single dose of MIS was 1 mg/g i.p. injection and assayed 24 h later. Two single doses were 1 mg/g on 1st day followed by 1 mg/g on 2nd day, and assayed 48 h after 1st injection.
Repeated injections were of a loading dose of 0·33 mg/g followed every 2 h by a maintenance dose of 0·16 mg/g, continued for 24 or 48 h before assaying.
Blood concentrations ranged from 60 to 118 g/ml.
The SF reported is an average of 3 experiments, each of 3 animals per point.
† The product of SF due to MIS and 10 Gy.

FIG. 2.—Surviving fraction of Lewis lung tumour cells exposed for various times *in vitro* to MIS at 100 μg/ml at 37°C, pH 7·4. ■ MIS in air; ● MIS in $N_2$; □ air only; ○ $N_2$ only.

reduction of cell survival. No cytotoxicity to oxic cells (under air) was detected.

### DISCUSSION

The half life of MIS in C57/BL mice reported above is considerably longer than the 1·0–1·5 h quoted by Flockhart (1978) for WHT mice.

The longer half life should be an advantage for *in-vivo* cytotoxicity experiments.

The results reported in Fig. 1b indicate rapid degradation of MIS in various tissues which were excised and left at room temperature. This was particularly rapid in liver. Drug degradation after removal from the host makes it necessary to assay or freeze tissues immediately after removal in order to obtain accurate estimates of concentration.

Many factors influence the cytotoxicity of nitroimidazoles. Hypoxia appears to be necessary for cytotoxicity to be observed. Sutherland (1974) has demonstrated preferential killing of the central cells in an *in-vitro* spheroid system. Preferential hypoxic-cell toxicity has been demonstrated *in vitro* by Stratford & Adams (1977) using Chinese hamster cells, by Courtenay (personal communication) and in the present work (Fig. 2).

Cytotoxicity is thought to be dependent on the metabolic alteration of MIS in contrast to radiosensitization, in which the unaltered drug is active in fixing radiation damage. This metabolic alteration into toxic metabolites probably occurs only under hypoxic conditions, and these metabolites may be able to kill both hypoxic and oxic cells. For example, *in-vitro* cytotoxicity to oxic cells can be demonstrated when these cells are exposed to the supernatant from hypoxic cells incubated with MIS (Whitmore *et al.*, 1978). *In-vivo* cytotoxicity to both hypoxic and oxic cells has been demonstrated in the present experiments (Table). Up to 50% cytotoxicity was observed, which is about twice the radiobiological hypoxic fraction in Lewis lung tumour observed by Shipley *et al.* (1975). Furthermore, the fraction of cells killed was similar to that in both the irradiated and unirradiated tumours. These observations suggest that *in-vivo* cytotoxicity is not selective to hypoxic cells, a conclusion also reached by Brown (1977) and Whitmore *et al.* (1978). Whitmore's results suggest that hypoxic cells reduce the MIS nitro group, forming one or more products which are toxic to all cells.

The concentration of MIS achieved in tumour cells is important, since the cytotoxic effect increases with concentration. Generally, however, the time course of cell exposure to drug is more critical than drug concentration. This has been shown *in vitro* by Stratford & Adams (1978) and in the *in-vitro* and *in-vivo* experiments reported in this paper. The tumours in mice receiving a single dose (1 mg/g) achieved a higher concentration of MIS but with a shorter exposure time than those in mice receiving multiple doses, and showed no apparent cytotoxic decrease in surviving fraction. A significant cytotoxicity (50%) was observed when the exposure time was increased by multiple dosage, despite a decrease in drug concentration in the tumour.

Hyperthermia (41–42°C) significantly enhances the cytotoxic effect of MIS *in vitro* (Stratford & Adams, 1977), whilst lowering the temperature below 37°C may provide the tumour cells with some protection. Mouse temperatures are very unstable, and will readily decrease when the animals are manipulated or given certain drugs, including MIS. The tumour temperatures in these experiments were often 2–3°C below normal core temperatures, which could be one explanation why little cytotoxicity was observed. Since temperature is an important factor in cytotoxicity, it may be possible to use hyperthermia clinically to enhance the cytotoxicity of MIS.

In conclusion, these cytoxic experiments demonstrate *in-vivo* cytotoxicity in the Lewis lung tumour system with concentrations which can be achieved in man, and when contact time is prolonged to 24 or 48 h. However, it is not possible to conclude that this cytotoxicity is selective for hypoxic cells, nor do these experiments contradict the necessity for hypoxic cells to be present for cytotoxicity to occur.

The authors acknowledge the help and advice of Dr G. G. Steel, Prof. G. E. Adams, Dr I. J. Stratford, Dr E. M. Fielden, Dr T. R. Stephens and Mr J. Peacock.

This work was supported by an R. S. McLaughlin Fellowship (JEP), the NCI (MRS) and the MRC/CRC (RDB).

### BIBLIOGRAPHY

Adams, G. E. (1977) Hypoxic cell sensitizers for radiotherapy. In *Cancer: A Comprehensive Treatise*, Vol 6. Ed. F. F. Becker. N.Y.: Plenum Press. p. 181.

Brown, J. M. (1977) Cytotoxic effects of the hypoxic cell radiosensitizer, Ro-07-0582 to tumour cells *in vivo*. *Radiat. Res.*, **72**, 469.

Courtenay, V. D. (1976) A soft agar colony assay for Lewis lung tumour and B16 melanoma taken directly from the mouse. *Br. J. Cancer*, **34**, 39.

Flockhart, I. R., Large, P., Troup, D., Malcolm, S. L. & Marten, T. R. (1978) Pharmacokinetics and metabolic studies of the hypoxic cell radiosensitizer misonidazole. *Xenobiotica*, **8**, (2) 97.

Hall, E. J. & Roizin-Towle, L. (1975) Hypoxic sensitizer; Radiobiological studies at the cellular level. *Radiology*, **117**, 453.

Kane, P. O. (1961) Polarographic methods for the determination of two antiprotozoal nitroimidazole derivatives in materials of biological and non-biological origin. *J. Polarogr. Soc.*, **7**, 58.

Shipley, W. U., Stanley, J. A. & Steel, G. G. (1975) Tumour size dependence in the radiation response of the Lewis lung carcinoma, *Cancer Res.*, **35**, 2488.

Steel, G. G., Hill, R. E. & Peckham, M. J. (1978) Combined radiotherapy–chemotherapy of Lewis lung carcinoma. *Int. J. Radiat. Oncol. Biol. Phys.*, **4**, 49.

Stratford, I. J. & Adams, G. E. (1977) The effect of hyperthermia on the differential cytotoxicity of the hypoxic cell radiosensitizer Ro-07-0582 on mammalian cells *in vitro*. *Br. J. Cancer*, **35**, 307.

Stratford, I. J. & Adams, G. E. (1978) The toxicity of the radiosensitizer misonidazole towards hypoxic cells *in vitro*: a model for man and mouse, *Br. J. Radiol.*, **51**, 745.

Sutherland, R. M. (1974) Selective chemotherapy of non-cycling cells in an *in vitro* tumour model. *Cancer Res.*, **34**, 3501.

Whitmore, G. F., Gulyas, S. & Varghese, A. J. (1978) Sensitizing and toxicity properties of misonidazole and its derivative. *Br. J. Cancer*, **37**, (Suppl III), 115.

Workman, P., Little, C. J., Marten, T. R., Dale, A. D., Ruane, R. J., Flockhart, I. R. & Bleehen, N. M. (1978) Estimation of the hypoxic cell sensitizer misonidazole and its O-demethylated metabolite in biological materials by reversed-phase high performance liquid chromatography. *J. Chromatogr.*, **145**, 507.